# Immediate Effects of a Novel Preterm Positioning Device on Physiological Stability in a Low Birth Weight Preterm Neonate: A Case Report

**DOI:** 10.7759/cureus.115299

**Published:** 2026-08-27

**Authors:** Dhwani D Chanpura, Gargi Patel

**Affiliations:** 1 Physiotherapy in Pediatric and Neonatal Sciences, College of Physiotherapy, Sumandeep Vidyapeeth (Deemed to be University), Vadodara, IND; 2 Physiotherapy, College of Physiotherapy, Sumandeep Vidyapeeth (Deemed to be University), Vadodara, IND

**Keywords:** low birth weight, neonatal intensive care unit, oxygen saturation, physiological stability, positioning device, preterm neonate, therapeutic positioning

## Abstract

Preterm infants admitted to the neonatal intensive care unit often require supportive positioning to promote comfort, physiological stability, and appropriate postural alignment. This case report describes the immediate physiological response of a medically stable low birth weight preterm infant during a single 120-minute positioning session using a novel preterm positioning device. A male infant born at 35 weeks of gestation was positioned in the supine position after achieving clinical stability at 37 weeks of gestation, and heart rate, respiratory rate, and peripheral oxygen saturation were monitored throughout the session. The infant remained clinically stable, with all physiological parameters staying within normal limits and no adverse events observed. The positioning device was well tolerated during routine care and appeared to support physiological stability. Although these findings are encouraging, further research involving larger groups of preterm infants is needed to determine the clinical effectiveness and longer-term benefits of this positioning approach.

## Introduction

Preterm birth remains a major contributor to neonatal morbidity and mortality worldwide. Despite advances in neonatal intensive care, maintaining physiological stability in preterm infants continues to be challenging because of the immaturity of their respiratory, cardiovascular, and neurological systems. Even routine handling and caregiving procedures can influence heart rate, respiratory rate, and oxygen saturation, emphasizing the need for developmentally supportive interventions that minimize stress while promoting physiological stability [1,2].

Therapeutic positioning is an important component of developmental care in the neonatal intensive care unit (NICU). Appropriate positioning helps maintain a flexed midline posture, enhances comfort, improves cardiorespiratory function, and supports neuromuscular development. Several studies have reported that different positioning strategies can positively influence physiological parameters and behavioral organization in preterm infants [2-5]. However, evidence regarding the effectiveness of novel positioning devices designed specifically for preterm infants remains limited.

The novel preterm positioning device used in this case was developed to provide supportive positioning for preterm and low-birth-weight infants by facilitating a flexed, midline, and developmentally appropriate posture. The device is made of medical-grade memory foam with a cotton covering and incorporates supportive components for the head and neck, shoulders, and lower limbs, with adjustable Velcro attachments to allow individualized positioning. Its design aims to provide gentle external support while allowing spontaneous limb movements. In neonatal care, such positioning support may be considered for medically stable preterm and low-birth-weight infants during developmental care, rest, routine handling, and other periods when maintenance of organized flexed alignment is desirable. The device is intended as an adjunct to standard neonatal care and does not replace clinical monitoring, respiratory support, or other medically indicated interventions.

Our research group previously developed a novel preterm positioning device and demonstrated its usefulness in improving postural development and caregiver satisfaction among preterm neonates admitted to the NICU [6]. Building on this work, the present case report describes the immediate physiological response of a medically stable low birth weight (LBW) preterm neonate following a single 120-minute session of supine positioning using the novel device. Heart rate (HR), respiratory rate (RR), and peripheral oxygen saturation (SpO_2_) were monitored to evaluate the infant's physiological tolerance to the intervention.

## Case presentation

A male preterm neonate born at 35 weeks of gestation by lower segment cesarean section (LSCS) was admitted to the NICU because of prematurity and LBW (989 g). The intervention was performed two weeks after birth, when the infant had attained a corrected gestational age of 37 weeks. At the time of assessment, the neonate was medically stable and was receiving enteral nutrition through a Ryle's tube.

The mother, a 35-year-old primigravida (G1P1A0L1), had attended regular antenatal check-ups and received routine nutritional supplementation during pregnancy. Antenatal ultrasonography showed no congenital abnormalities, and there was no history of maternal hypertension, diabetes mellitus, trauma, addiction, or other significant antenatal complications. The infant was delivered by LSCS with an immediate cry after birth and was admitted to the NICU because of prematurity and LBW. There was no history of ventilatory support, supplemental oxygen therapy, neonatal seizures, significant jaundice, or congenital anomalies. Family history was unremarkable.

Clinical examination before the intervention revealed a hemodynamically stable neonate with normal skin color, an appropriate level of consciousness, and spontaneous symmetrical limb movements. Respiratory examination demonstrated regular abdominal breathing with equal bilateral air entry and no added sounds. Primitive reflexes, including palmar grasp, plantar grasp, Moro reflex, placing reflex, and Galant's reflex, were present, while rooting, sucking, primary walking, and extensor thrust were weak, consistent with prematurity. Muscle tone and posture were appropriate for the infant's gestational age.

**Figure 1:**
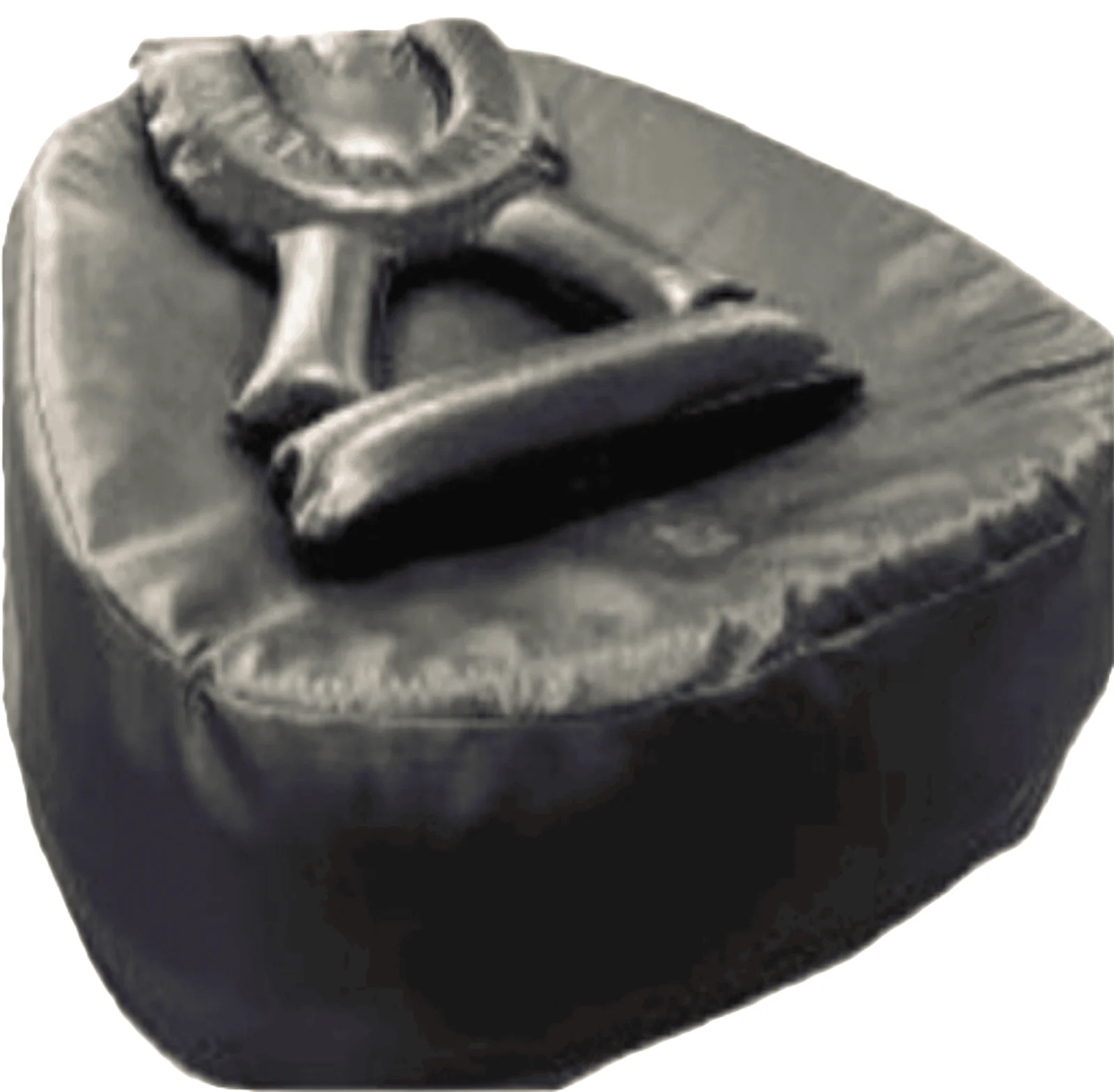
Preterm positioning device This novel preterm positioning device is protected by a registered design patent (Design Number: 403807-001)

**Figure 2:**
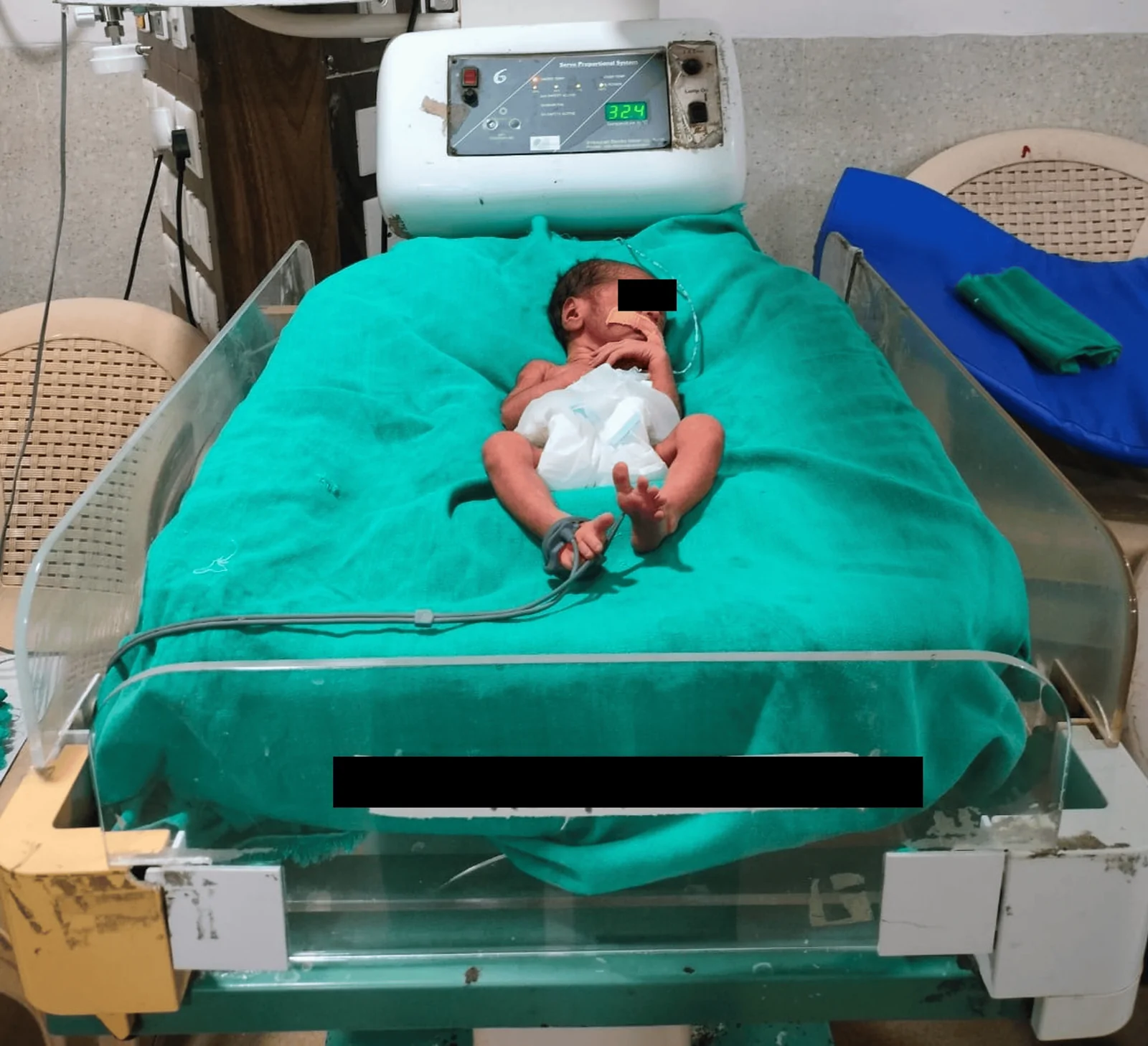
Supine positioning of a preterm infant using the novel preterm positioning device during the intervention session.

The infant was positioned in the supine position using a novel preterm positioning device developed to provide supportive postural alignment for preterm and LBW infants (Figures 1, 2). The device is constructed using medical-grade memory foam with a cotton covering and includes supportive components for the head and neck, shoulders, and lower limbs. Adjustable Velcro attachments permit the supports to be adapted to the infant's body size and positioning needs. The design aims to facilitate a flexed, midline posture and provide gentle external support without unnecessarily restricting spontaneous movements. In this case, the device was used as an adjunct to developmental positioning in a medically stable infant; no additional physiotherapy intervention was administered during the observation period.

At baseline, the infant's HR was 162 beats/minutes, RR was 45 breaths/minute, and SpO₂ was 95% (Table 1). After 60 minutes, HR decreased to 152 beats/minute, RR to 42 breaths/minute, and SpO₂ was 95%. At the completion of 120 minutes, HR was 165 beats/minute, RR was 43 breaths/minute, and SpO₂ improved to 96%. The infant tolerated the intervention well without episodes of apnea, bradycardia, feeding intolerance, or any other adverse events.

**Table 1:** Physiological parameters recorded during the 120-minute positioning session.

Time point	Heart Rate (beats/min)	Respiratory Rate (breaths/min)	Peripheral Oxygen Saturation (%)
Baseline	162	45	95
60 minutes	152	42	95
120 minutes	165	43	96

The intervention was well tolerated, and physiological parameters remained within an acceptable clinical range throughout the observation period, supporting the feasibility and safety of using the novel positioning device in a medically stable preterm neonate. Because this was a single-case observation, the physiological values were interpreted descriptively. The small changes in HR, RR, and SpO₂ were not interpreted as evidence of clinical improvement or treatment efficacy.

## Discussion

Therapeutic positioning is an integral component of developmental care in the NICU, aiming to promote physiological stability, improve comfort, and support neuromuscular development in preterm infants [1]. Appropriate positioning helps maintain a flexed midline posture, reduces energy expenditure, and facilitates cardiorespiratory regulation, which is particularly important in premature neonates with immature physiological systems [7]. These observations indicate that, in this single medically stable infant, the device did not result in clinically apparent physiological destabilization during the 120-minute observation period.

In the present case, the 35-week preterm neonate tolerated 120 minutes of supine positioning using a novel preterm positioning device without any adverse events. HR and RR remained within an acceptable clinical range throughout the intervention, while SpO_2_ improved from 95% at baseline to 96% at the end of the observation period. These findings suggest that the device can be safely used during routine NICU care without compromising physiological stability.

Previous studies have also reported favorable physiological responses to developmental positioning in preterm infants. Santos et al. demonstrated that structured positioning contributed to stable physiological parameters and improved behavioral organization [2], while Cheraghi et al. observed improved oxygen saturation with therapeutic positioning [3]. Although these studies evaluated conventional positioning methods, the present case indicates that a customized positioning device designed to maintain flexed alignment may provide similar physiological support.

Our research group (Chanpura et al.) has previously reported that this novel positioning device improved postural development and caregiver experiences in preterm neonates receiving physiotherapy interventions [6]. The present case complements those findings by demonstrating that the device was well tolerated during a single positioning session and maintained physiological stability in an LBW preterm infant. However, as this report describes only a single case, larger prospective studies are required to establish its clinical effectiveness and generalizability.

Limitations

This report has several limitations. First, it describes a single infant and a single 120-minute positioning session; therefore, the findings cannot establish efficacy or generalizability. There was no control or comparison period in standard positioning, so changes in physiological parameters cannot be attributed specifically to the device. The infant was already medically stable before the intervention, and physiological variability related to handling, feeding, time of day, and other routine NICU factors may have influenced the observed measurements. The observation was limited to supine positioning and one LBW male preterm infant, which limits extrapolation to other gestational ages, birth weights, sexes, clinical conditions, or positioning strategies. In addition, the present case assesses short-term tolerance only and does not evaluate long-term developmental outcomes, device-related skin effects, or comparative effectiveness against conventional positioning aids. Larger controlled studies with standardized comparison conditions and longer follow-up are required to evaluate the safety, effectiveness, and generalizability of the device.

## Conclusions

This case demonstrates that the novel preterm positioning device was well tolerated during a single 120-minute supine positioning session in a medically stable LBW preterm neonate, without clinically apparent physiological destabilization or adverse events. However, the findings cannot establish efficacy or causality. Larger controlled studies are required to determine the safety, effectiveness, and long-term clinical benefits of the device.
